# Clinical implication of device-based algorithm that optimize atrioventricular delay during cardiac resynchronization therapy: author’s reply

**DOI:** 10.1007/s00380-022-02205-w

**Published:** 2022-11-27

**Authors:** Yoshifumi Ikeda, Ritsushi Kato

**Affiliations:** Department of Cardiology, Saitama Medical University International Medical Center, 1397-1 Yamane, Hidaka, Saitama, 350-1298 Japan

We really appreciate Dr. Kataoka and Dr. Imamura for their interest and important suggestion to our manuscript [1]. As they mentioned, we acknowledge that there are many factors affecting to the optimal AV delay such as activity, exercise, heart rate, the timing of LV inflow patterns and so on. We also understand that many factors such as severity and type of electronical and mechanical dyssynchrony, scar burden and pacing sites are associated with the response for the CRT. It has been well known there are still about 30% non-responder after the CRT. Optimization of AV delay is one of the important methods to improve the response of CRT. Although the echocardiographic evaluation is a gold standard to adjust the AV delay, only a few physicians have been repeatedly performing this method because of time consuming. However, repeated adjustment of AV delay is very important for the clinical outcome after the CRT implantation as we showed in the manuscript. Therefore, many device-based algorithms (DBAs) have been developed to overcome the weakness of the evaluation of echocardiography, and non-inferiority in the clinical outcome compared with echo-guided method has been shown previously in most of those DBAs [2–5].

An optimal AV delay using “SmartDelay™ “ and “SyncAV™” which are the relatively new DBAs is calculated according to patients’ own AV conduction time. While, “QuickOpt™”, which was mainly used in this study, is an algorithm to obtain an optimal AV delay from the atrial wave duration time by the uni-polar of the atrial lead independently of patients’ own AV conduction time. “AdaptiveCRT™” (aCRT) is an algorithm that can provide ambulatory adjustment of AV delay, however, it behaves like QuickOpt if the patients’ own AV conduction time is too long or blocked. Therefore, QuickOpt or aCRT can be used even in the patients with AV block. We used those two algorithms in 77% (47/61) of patients in this study, and we believe that the influence by differences in patients’ own AV conduction time was minimum. Fortunately, the baseline PR interval between the two groups excluding AVB was statistically equivalent. (Group 1 vs Group 2; 193.9 ± 79.3 vs 191.2 ± 34.1 ms, *p* = 0.77). We also found there was no significant differences between the groups in terms of the prevalence of the AV block (Group 1 vs Group 2; 16% (9 patients) vs 25% (14 patients), *p* = 0.18) and age, characteristics of disease shown in the manuscript, as well.

Because of the study design, we did not use the echocardiography for the adjustment of AV delay in this study and we have not known well the best adjusting method for optimal AV delay depending on the age, disease and dependency of pacemaker, as Dr. Kataoka and Dr. Imamura mentioned. Future study using the DBAs with continuous hemodynamic parameters such as contractility sensor or other monitors instead of electrocardiographic parameters for many kinds of patient background may provide the answer for those questions [6].

## Data Availability

This study has not been registered in an data repository. Therefore our data are not publicly available in the Internet. However, the datasets generated and/or analyzed during the current study are available from the corresponding author on reasonable request.
